# The Comparative Full-Length Genome Characterization of African Swine Fever Virus Detected in Thailand

**DOI:** 10.3390/ani14172602

**Published:** 2024-09-06

**Authors:** Muhammad Salman, Dhithya Venkateswaran, Anwesha Prakash, Quynh Anh Nguyen, Roypim Suntisukwattana, Waranya Atthaapa, Angkana Tantituvanont, Tapanut Songkasupa, Taweewat Deemagarn, Kultyarat Bhakha, Nuttun Pengpetch, Janya Saenboonrueng, Theeradej Thaweerattanasinp, Anan Jongkaewwattana, Dachrit Nilubol

**Affiliations:** 1 Swine Viral Evolution and Vaccine Development Research Unit, Department of Veterinary Microbiology, Faculty of Veterinary Science, Chulalongkorn University, Bangkok 10330, Thailand; 2 Department of Pharmaceutic and Industrial Pharmacies, Faculty of Pharmaceutical Sciences, Chulalongkorn University, Bangkok 10330, Thailand; 3 Department of Livestock Development, National Institute of Animal Health, 50/2 Kasetklang, Phahonyothin 45-15, Chatuchak, Bangkok 10900, Thailand; 4 National Center for Genetic Engineering and Biotechnology, Pathum Thani 12120, Thailand

**Keywords:** African swine fever virus (ASFV), genotype, serotype, IGR, TRS, P72

## Abstract

**Simple Summary:**

African swine fever is a deadly disease affecting pigs worldwide, causing major economic losses. The virus strain causing the current outbreak in Europe and Asia, known as genotype II, was first identified in Georgia in 2007. This study aimed to analyze the genetic makeup of a strain found in Thailand to better understand its characteristics and how it compares to other strains in Asia. The viral DNA from an infected pig was analyzed and was found to be almost identical to strains from Georgia and China. The Thai strain had eight unique genetic changes, including small mutations in specific genes and deletions in others. This detailed genetic analysis helps track the spread and evolution of the virus, providing important information for developing strategies to control future outbreaks. By understanding these genetic differences, improved measures to protect pig populations can be implemented, thereby reducing the economic impact on the agricultural industry.

**Abstract:**

African swine fever virus (ASFV) has been responsible for the globally devastating epidemics in wild and domesticated pigs. Of the 24 identified ASFV genotypes, genotype II is the primary cause for the pandemic occurring in Europe and Asia since its emergence in Georgia in 2007. The current study aimed to characterize the full-length genomic pattern of the ASFV strain from Thailand, TH1_22/CR (Accession No. PP915735), which was then compared with genomic diversity across other Asian isolates using Georgia 2007/1 (Accession No. FR682468) as the reference. Viral DNA was isolated from the pig spleen sample following library preparation and paired-end sequencing using the MiSeq Illumina platform. The sequenced TH1_22/CR isolate spanned 189,395 nucleotides encoding 193 open reading frames (ORFs), exhibiting maximum nucleotide similarity (99.99%) with Georgian (Georgia 2007/1) and Chinese (Wuhan 2019-1 and China HLJ) isolates. Based on phylogenetic analysis, the TH1_22/CR isolate (Accession No. PP915735) was characterized as genotype II, serogroup 8, and IGR-II due to the presence of three tandem repeat sequences (TRSs). Genetic variations including SNPs and single and polynucleotide indels were identified in TH1_22/CR in agreement with other Asian isolates. For comprehensive analysis, the genome was divided into four regions (I–IV) based on gene location. Overall, the TH1_22/CR isolate demonstrated eight SNPs and indels in its genome. Two unique SNPs were reported in the coding regions of the TH1_22/CR isolate, out of which, a C-591-T substitution was seen in MGF 360-4L and a C-297-T was found in A238L, and four unique SNPs were reported in non-coding regions (NCRs). Furthermore, a 29 bp deletion was observed in the IGR between MGF 110-13La and MGF 110-13Lb, as well as 52 bp deletion in the ASFV G ACD 00350 gene. This comparative analysis establishes the foundational information for future studies on the diversity and phylogeography of this regionally significant genetic sub-group of ASFV.

## 1. Introduction

African swine fever virus (ASFV) is the causative agent of African swine fever (ASF), a highly contagious hemorrhagic disease. Depending on the virulence of the ASFV strain, the clinical presentation varies from mild fever and respiratory distress in low virulent strains to acute fever and sudden death in highly virulent strains. Anorexia and lethargy are common presentations in the infection of all ASFV variants [1]. ASFV solely belongs to the family *Asfarviridae* under the genus *Asfivirus* and impacts domestic and wild suids of all ages with a mortality rate nearing 100 percent in peracute infections caused by highly virulent strains [2]. Using the partial nucleotide sequence in the C-terminal end of the B646L gene which codes for the p72 capsid protein, ASFV strains have been classified into 24 genotypes [3,4]. Based on these 24 genotypes, all non-African ASFV field strains are classified as genotypes I and II [5].

The genomic content of this double-stranded DNA virus is in the range of 170–194 kilobases comprising 151–186 open reading frames (ORFs) [6,7]. Hypothetically, the viral genome is divided into a left variable region (LVR), a right variable region (RVR), and a central variable region (CVR). The LVRs and RVRs display high diversity, especially in the multi-gene family (MGF) genes. The LVR contains important genetic determinants that impact the virulence, replication features, and host range functions of the virus. In the complete genome of Georgia 2007/1 (FR682468.2), the total number of MGF genes is 45, which can be classified into five MGF types. The most abundant MGF is MGF-360 (n = 19), followed by MGF-505 (n = 10), MGF-110 (n = 10), and MGF-300 (n = 3). Out of all MGF-360 genes, 14 are in the LVR and 5 in the RVR. In the case of MGF-505, nine genes are found in the LVR, and the remaining one gene is in the RVR. Finally, all MGF-110 and MGF-300 genes exclusively occur in the LVR. The B602L gene is reported as the CVR region, which is highly informative in understanding the genetic diversity, classification, and evolution of the ASFV strains circulating in various territories [8,9].

On the contrary, the central conserved region (CCR) is an evolutionarily stable region comprising replication-associated genes with conserved structural and enzymatic functions that play a role in the viral assembly and modulation of host-cell functions. Such genes reflect the essential requirements for the cytoplasmic replication of a complex system. This approach has yielded valuable insights for classifying specific ASFV isolates, as highlighted in a previous report [9]. The CCR encompasses genes associated with viral replication, assembly, and modulation of host-cell functions. Although the derived data are subject to constraints and may not conclusively elucidate the provenance of closely affiliated viral strains, molecular markers such as viral genes (E183L, B602L, KP86R, I196L, and EP402R) and the intergenic regions (IGRs) of I73R/I329R and I78R/I215L have also been employed to discriminate closely related strains within a shared genotype [10].

ASF was initially identified in 1921 in Kenya [11]. Following this, the disease advanced to various sub-Saharan African countries [12] and became endemic across Africa. The strain, assigned to ASFV genotype I, subsequently spread into Europe, South America, and the Caribbean [13]. Fortunately, this genotype has been eliminated from these regions as a result of effective measures. In contrast, however, genotype II of this virus, which emerged in Georgia in 2007, has remained widespread in Europe and Asia, leading to severe global economic impacts on the pig industry. This genotype has exhibited a notable capacity for transboundary transmission through indirect routes like contaminated pork products and the flux of infected animals and fomites [14,15].

In China, the first case of ASF was reported in June 2018. Subsequently, ASF outbreaks have rapidly spread across neighboring Asian countries within a short span of 9 months, endangering the substantial domestic pig population of over 1.2 billion [16,17,18]. Currently, ASFV outbreaks have been documented in several countries across Southeast Asia [19,20,21]. In 2022, Thailand officially announced its first case of ASF [22].

Since ASFV in Thailand has only been detected but not described so far, this study provides a novel comprehensive analysis of the full-length ASFV genome sequence of TH1_22/CR (Accession No. PP915735), a strain representing an outbreak in Thailand. By comparing the variations observed in the genes of neighboring regions, this study also highlights the similarity of the Thai isolate with the Georgia 2007/1 strain and other strains selected from Asian countries like China, Vietnam, Korea, and the Philippines, which were chosen for analysis based on the geographical distance or time of the outbreak. Therefore, our study aims to understand the evolutionary identity of TH1_22/CR.

## 2. Materials and Methods

### 2.1. Sampling and DNA Extraction

Spleen samples from 6-month-old pigs were submitted to the National Institute of Animal Health for whole genome sequencing using next-generation sequencing (NGS). The genome sequences were determined directly from the clinical samples to avoid cell-culture-driven sequence variation as reported previously [17]. The genomic DNA of ASFV was extracted from the sample following the protocol of the DNeasy blood and tissue kit (Qiagen, Hilden, Germany). To eliminate methylated swine DNA contaminants in the ASFV DNA samples, a NEBNext^®^ Microbiome DNA Enrichment kit (New England Biolabs, Ipswich, MA, USA) was employed according to the manufacturer’s guidelines. Subsequently, the quality of the DNA was assessed through agarose gel electrophoresis and the purity of the DNA was evaluated via the absorbance ratio at 260/280 nm using a Nanodrop spectrophotometer (ThermoFisher Scientific, Waltham, MA, USA). The concentration of DNA was quantified using a Qubit fluorometer with a dsDNA High Sensitivity assay kit (Invitrogen, Carlsbad, CA, USA). The obtained isolated DNA was then stored at −20 °C until further processing.

### 2.2. DNA Library Preparation and High-Throughput Sequencing

High-quality isolated DNA (1 µg) was used to construct a library using a Nextera XT DNA library preparation kit (Illumina, San Diego, CA, USA) according to the manufacturer’s instructions. In brief, DNA was fragmented into approximately 300 bases by transposons, followed by adapter ligation and few-cycle amplification. The amplified library was purified with Agencourt magnetic beads (Beckman Coulter, Brea, CA, USA). The concentration of the library was quantified using the dsDNA High Sensitivity assay Qubit kit and the quality of the library was evaluated using an Agilent 4200 Tape Station system with a High Sensitivity D1000 Screen Tape assay kit (Agilent Technologies, Santa Clara, CA, USA). The library was denatured and diluted to a final concentration of 16 pmole for high-throughput sequencing. Paired-end sequencing was performed on a v2 flow cell with 2 × 300 cycle sequencing chemistry (Illumina, San Diego, CA, USA). The quality of the obtained sequencing data was evaluated using the FastQC v. 0.12.1 tool [23]. The adapter sequences and low-quality reads were removed by the BBDuk Trimmer tool (https://jgi.doe.gov/data-and-tools/software-tools/bbtools/bb-tools-user-guide/bbduk-guide/, accessed on 2 January 2024). Good quality sequencing short reads were processed for de novo assembly using SPAdes v. 3.15.2. The quality of the assembled genome was assessed by the QUAST tool. A BLAST analysis of the assembled genome was performed using the BLASTn method and the best-matched sequence was selected as the reference genome.

### 2.3. Characterization of TH1_22/CR

The ASFV was characterized based on genotyping using the p72 protein, serogrouping by the CD2v protein, and tandem repeat sequences (TRSs) [24]. A specific set of primers were used as described by earlier studies for the amplification of the C-terminal region of the B646L gene which encodes for p72 [3], the complete EP402R gene encoding for the CD2v protein [25], the B602L gene which contains the CVR [26], and the TRS located between the I73R and I329L genes [27]. All amplified products were processed with Sanger sequencing followed by BLAST analysis. The sequences were processed for pairwise multiple alignments of the p72 and CD2v regions with reference sequences followed by a phylogenetic tree construction using MEGA [28].

All sequence alignments and phylogenetic analyses were performed using MEGA 7 [28]. A neighbor-joining (NJ) tree of the p72 nucleotide sequences was constructed using the maximum likelihood composite method. For the CD2v gene, a maximum likelihood (ML) tree of the partial amino acid sequences was constructed by applying the LG model [29]. The bootstrap method was used to re-sample the data 1000 times for the NJ and ML trees. The final tree was midpoint rooted using FigTree v1.4.2 for comprehensive evolutionary relationships (https://tree.bio.ed.ac.uk/software/fgtree/, accessed on 12 January 2024). For the CVR profile analysis, the partial pB602L gene sequence of the TH1_22/CR strain was translated, and the resulting amino acid tetramers were matched with previously reported sequences [30].

### 2.4. Retrieval of Public ASFV Genome Sequences and Alignment

A total of 29 publicly available complete genomes of genotype II ASFV strains from Asian countries were retrieved from the NCBI database on 15 January 2024, along with our in-house sequenced TH1_22/CR strain, to construct a phylogenetic tree for the whole genome. The Thai isolate of ASFV-TH1_22/CR was submitted to GenBank (Accession No. PP915735). In the strain designation TH1_22/CR, each component has a specific meaning: “TH1” denotes Thailand, “22” represents the year 2022 when this strain was isolated, and “CR” stands for Chiang Rai in Thailand, the province from which this strain was collected. The Asian strains originate from 5 countries: China (n = 16), the Philippines (n = 5), Korea (n = 5), Vietnam (n = 2), and Timor Leste (n = 1) (Table 1). Since genotype II originates from Georgia, the Georgia 2007/1 strain (accession number FR682468.2) was used as the reference throughout the study. Information, including genotype, country, host, sampling location, and collection date, was acquired from the GenBank records or relevant publications. The viral genomic data were downloaded in the FASTA format from GenBank. Post-phylogenetic tree construction, 8 out of the 31 strains were chosen for the similarity index and variation analysis of TH1_22/CR. The selected strains are from China [Wuhan 2019-1 (MN393476) and Pig/HLJ/2018 (MK333180)], the Philippines [PHL/Strain A4 (ON963982) and ASFV2019-003-B (MW791760)], Korea [Korea/pig/Yeoncheon1/2019 (MW049116) and S-S-VR-413000-00008 (OR159218)], and Vietnam [VN/2019 (MT882025) and VN/2021(ON402789)]. Therefore, 2 isolates from each country were selected based on the criteria of a notable outbreak, with either significant geographical distances or from different years (if a country has major outbreaks exclusively from a particular location). The average nucleotide identity (ANI) of these strains and TH1_22/CR, along with the reference Georgia 2007/1, was estimated using BioEdit software. All strains exhibiting an ANI more than 98% were processed for sequence alignment using fast multiple sequence alignment MAFFT software (v 7.475) as described earlier by [31].

### 2.5. Genetic Analysis

ASFV exhibits considerable genetic variation in ORFs and intergenic regions (IGRs). Single nucleotide polymorphisms (SNPs) are the most common evolutionary forms of genomic variation that can influence viral replication and pathogenesis. Intergenic regions play a critical role in regulating viral gene expression and may harbor sequences involved in host interactions or immune evasion mechanisms. Insertions and deletions (Indels) also cause variations in DNA where nucleotides are either added or removed from a genome, causing a shift in the sequence length. Based on SNPs and indels at the genomic level, the correlation between TH1_22/CR and other Asian isolates was investigated.

### 2.6. Analysis of ORFs for Sequence Diversity

The genome-wide diversity in ASFV was investigated using pairwise sequence similarity and the distribution of ORFs. The genomic sequences of the TH1_22/CR and Asian isolates were compared with Georgia 2007/1 as the reference strain.

### 2.7. Genetic Variation for SNPs and Single Indels

The Genome Annotation Transfer Utility (GATU) tool was employed to identify open reading frames (ORFs) in the genome. The minimum length threshold was set to 30 codons [32]. Comparative analysis of the sequence identity of each ORF in every strain was conducted to its corresponding ortholog in Georgia 2007/1.

### 2.8. Amino Acid Variation for SNPs and Single Indels

The DNA sequence was translated to amino acids using the BioEdit tool version 7.2.5) for identifying conservative and non-conservative mutations. In conservative mutations, an amino acid is replaced by a similar one, which preserves the structure and function of the protein. Non-conservative mutations replace amino acids with completely different properties and can significantly change the structure and function of the protein.

### 2.9. Phylogenetic Tree of the Whole Genome

The phylogenetic tree of the whole genome sequence was analyzed, which showed the evolutionary relationships between different isolates by comparing their entire genetic makeup. In the current study, the genomes of the Thai and Asian isolates were aligned using MAFFT software [33], and a neighbor-joining (NJ) phylogenetic tree was constructed using MEGA 11 software [28]. Using the bootstrap method, the data were re-sampled 1000 times for the NJ and ML trees. The final tree was midpoint rooted using FigTree v1.4.2 for comprehensive evolutionary relationships (https://tree.bio.ed.ac.uk/software/fgtree/, accessed on 13 February 2024). To understand genetic similarities and differences, the phylogeny provided information about their evolutionary history and relationships.

### 2.10. Intergenic Region Analysis

The viral genome contains a specific intergenic region in which isolates show differences in their TRS, oligonucleotides, indels in the DNA fragments, and variations in the number of poly GC counts. This intergenic region exhibits genetic diversity between the different isolates, potentially affecting viral properties such as virulence, transmission, or susceptibility to treatment.

### 2.11. Tandem Repeat Sequence Variation

A tandem repeat sequence is a pattern found in DNA that is repeated directly next to itself on a chromosome. These repeats vary in length and are often found in non-coding regions of the genome. Tandem repeat sequences (TRSs) throughout the TH1_22/CR genome were detected utilizing the Tandem Repeats Finder or TRF program version 4.09 [8]. The chosen parameters for match, mismatch, and delta were 2, 7, and 7, respectively; the minimum score threshold was 50.

### 2.12. Oligonucleotide Indels

The intergenic region of the ASFV genome refers to the DNA segments between the ORFs of the virus. Oligonucleotides are short sequences of nucleotides that are usually more than 10 nucleotides long. Analyzing these sequences provides insights into potential regulatory elements, functional motifs, or genetic variations within the intergenic regions. This contributes to the understanding of ASFV gene expression, replication, and pathogenesis. The intergenic region of the ASFV genome was analyzed for oligonucleotides using BioEdit [34]. The variations in the other genomic sequences were subsequently analyzed.

## 3. Results

### 3.1. Whole Genome Sequence and Similarity Index of TH1_22/CR

**Figure 1:**
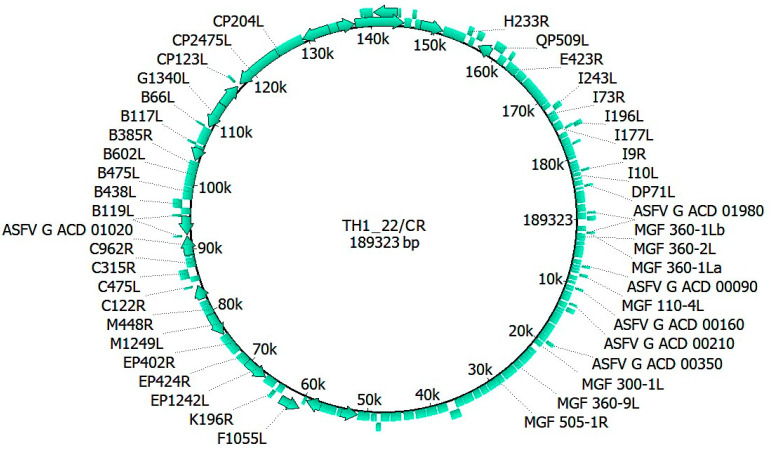
Circular genomic profile of the TH1_22/CR ASFV isolate. Positions of protein-coding genes are indicated by plain arrows and bars, with the direction of the arrows indicating the 5′ to 3′ orientation of the coding sequence. The numbers inside the circle show the length of the genome. The dotted lines specify the gene’s location, and these lines are mentioned with gene names.

In this study, we retrieved a total of 2,590,054 reads from the sequencing process. Of these, 64.32% were specific to the ASFV. The mean of the coverage for ASFV-specific sequences was 2.8421. The genome of TH1_22/CR was annotated as 189,323 base pairs (bp), which revealed 193 ORFs and 185 coding sequences. The GC content was evaluated as 38.18%. Compared to the sequence retrieved from the NCBI GenBank, our TH1_22/CR genome sequence was shorter than Georgia 2007/1 (190,584 bp), with a difference of 1261 base pairs. This discrepancy is attributed to the 5′ (956 bp) and 3′ (304 bp) overhanging ends/flanking regions in the Thai genome (Figure 1).

The average nucleotide identity (ANI) and amino acid similarity of TH1_22/CR with the other strains revealed a range of 99.1% to 99.9%. With the reference Georgia 2007/1, our strain exhibits a 99.9% ANI. For the other strains, it showed the highest similarity of 99.9% ANI to Pig/HLJ/2018 and Wuhan 2019-1 from China, PHL/Strain A4 and ASFV2019-003-B from the Philippines, and the S-S-VR-413000-00008 strain from Korea. The amino acid similarity was the same for the strains except for the PHL/Strain A4 and S-S-VR-413000-00008 strains (99.8%), indicating the presence of non-synonymous mutations in these strains. The least similarity was with Korea/pig/Yeoncheon1/2019 from Korea and VN/2021 from Vietnam. These strains showed a 99.1% and 99.2% nucleotide similarity, and a 99.1% and 99.2% amino acid similarity, respectively. These variations are not significantly different, clearly indicating a high degree of conservation across these strains. The similarity index is illustrated in Table 2.

### 3.2. Characterization of TH1_22/CR

**Figure 2:**
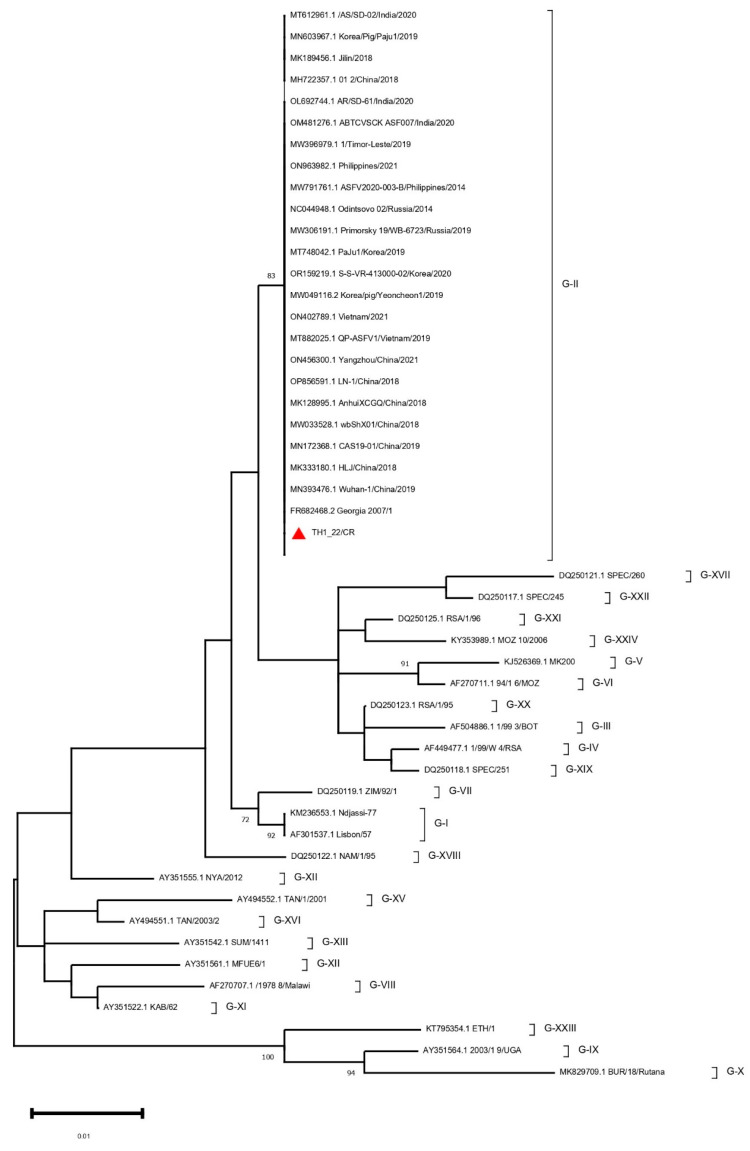
Maximum likelihood phylogenetic tree of ASFV based on P72 gene sequences in the curated dataset. The tree is midpoint rooted. Re-sampling with 1000 bootstrap replications was conducted. The red triangle indicates TH1_22/CR.

**Figure 3:**
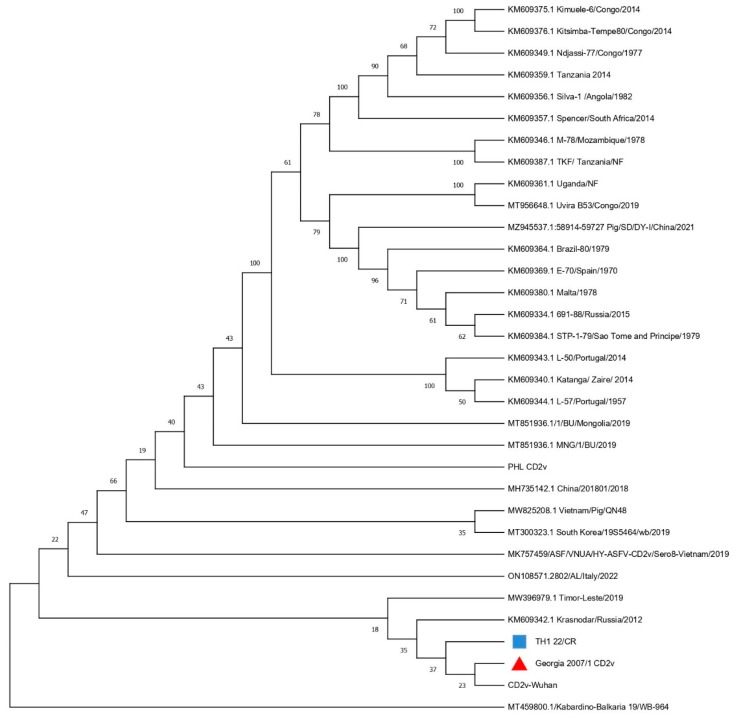
Neighbor-joining phylogenetic tree of ASFV based on CD2V gene sequences in the curated dataset. The tree is midpoint rooted. The maximum likelihood method with 1000 bootstrap replications was used. The blue square indicates the CD2V gene in TH1_22/CR and the red triangle indicates the CD2V gene in the Georgia 2007/1 strain.

The existence of ASFV in the sample was verified through traditional PCR using p72 primers, which amplified a 418 nucleotide-long fragment consistent with the anticipated size for ASFV. This sequence was used to construct a phylogenetic tree, which effectively classified the TH1_22/CR sequence. The analysis of the C-terminal end of the p72 gene of the Thai strain alongside 24 genotyping reference sequences confirmed that the Thai ASFV isolate belongs to genotype II (Figure 2). Serogrouping using the CD2v-coding EP402R gene sequence revealed that TH1_22/CR belongs to serogroup 8, evident from the clustering of this strain with other serogroup 8 ASFV isolates (Figure 3). The CVR analysis placed our strain in the CVR-I group due to the “BNDBNDBNAA” TRS profile, showing a total sequence identity to Georgia 2007/1.

### 3.3. Phylogenetic Tree of the Whole Genome of TH1_22/CR

**Figure 4:**
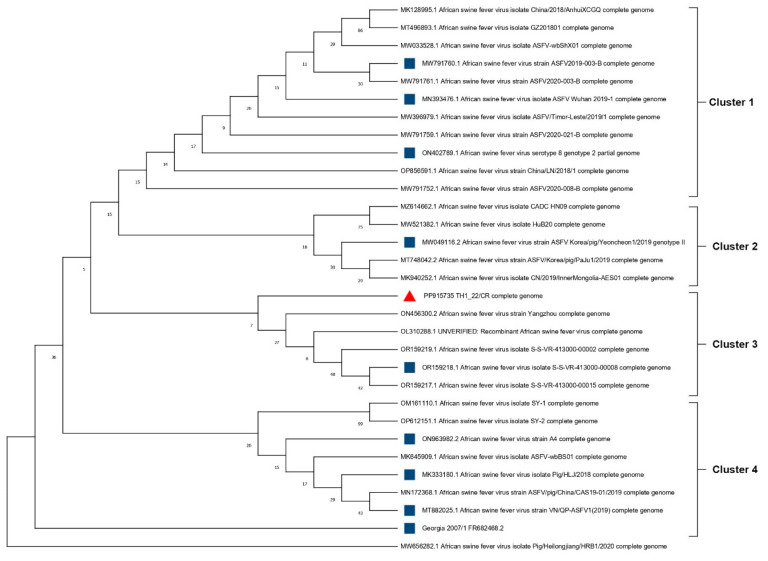
**Whole genome sequencing (WGS) phylogenetic tree.** The WGS phylogenetic tree was constructed using Georgia 2007/1 as the reference and the complete genome representatives of ASFV from Asia using the MEGA version XI software package. The phylogenetic tree containing 31 strains was re-constructed using the maximum composite likelihood method to compute the evolutionary distances. The statistical significance of the phylogenies constructed was estimated by a bootstrap analysis of 1000 replicate datasets. The red triangle indicates the whole genome of TH1_22/CR and the blue squares indicate the selected Asian whole genome ASFV isolates.

Using MEGA software version 11 and a GTR evolution model, an ML tree was constructed containing the complete genome sequencing data of our Thai sequence, the reference, and the 30 full-length ASFV genome sequences acquired from the GenBank. In this tree, TH1_CR/2022 is categorically classified as a part of the genotype II ASFV taxonomic unit. Four distinct clusters can be observed and TH1_22/CR lies in Cluster 3. Our strain is evolutionarily closer to the Yangzhou (ON456300.2) and UNVERIFIED (OL310288.1) strains from China, and the S-S-VR-413000-00002 (OR159219.1), S-S-VR-413000-00008 (OR159218.1), and S-S-VR-413000-00015 (OR159217.1) strains from Korea. Out of all the strains, Pig/Heilongjiang/HRB1/2020 (MW656282.1) appears to be significantly different (Figure 4).

### 3.4. Comparative Analysis of the Variations in TH1_22/CR

The Thai genome was compared with the Georgia 2007/1 genome, followed by the selected Asian strains. To understand the ASFV whole genome better, the genome of Georgia 2007/1 (FR682468) was divided into four regions i.e., Region I to Region IV. Region I, also referred to as the LVR, consists of 47,170 nucleotides (starting from the 5′ end) harboring 59 genes. Region II comprises 54,378 nucleotides (47,287 bp to 101,665 bp) with 49 genes. Region III has a total of 44 genes, which comprises 60,996 nucleotides of the genome (101,676 bp to 162,672 bp). Region IV, also called RVR, contains 27,549 nucleotides (162,620 bp to 190,169 bp) and is the last part of the genome harboring 43 genes (Table 3). Comparing these reference regions with the Thai isolate revealed nine variations in its genome.

### 3.5. Genetic Variations in the ORFs of TH1_22/CR

In TH1_22/CR, two unique SNPs, five shared SNPs, and one shared insertion were identified in ORFs in different regions. In Region I, the novel C-to-T mutation was observed at position 591 in MGF 360-4L (C-591-T). Further, our strain shares three SNPs with the other Asian strains in this region: a C-to-T substitution at the 51st position of MGF 110-1L (C-51-T), a T-to-C mutation at the 53rd position of MGF 360-10L (T-53-C), and an A-to-G substitution at the 962nd position of the MGF 505-9R gene (A-962-G).

In Region II, a unique C-to-T mutation can be identified at position 297 of the A238L gene. No shared mutations can be found in this region. Region III has one notable SNP: a C-to-T substitution at the 20th position in the NP419L gene (C-20-T). The isolate shares this substitution with the other Asian isolates considered.

Finally, our isolate shows two shared mutations in Region IV. One SNP is seen at position 222 of the I267L ORF carrying a T-to-A substitution (T-222-A). An adenine insertion can be observed at the 104th position of DP60R (X-104-A), which is shared with all the other strains except PHL/StrainA4 from the Philippines. Additionally, four substitutions can be observed in the non-coding region of TH1_22/CR, consisting of the A-to-G (A-189406-G), C-to-G (C-189433-G), G-to-A (G-189439-A), and T-to-A changes (T-189459-A) (Table 4).

### 3.6. Amino Acid Variations in TH1_22/CR

In the case of amino acids, two unique substitutions and six shared mutations can be seen distributed in the different regions of our strain. For Region I, the unique Ala to Thr non-conservative change at position 192 of the protein coded by the MGF 360-4L gene was noted (Ala-192-Thr). TH1_22/CR also has three shared mutations in this region which are shared between all the isolates; a non-conservative Pro to Leu mutation at the 19th position of pMGF-110-1L (Pro-19-Leu), a conservative Val to Ala change at the 18th position of pMGF 360-10L (Val-18-Ala), and a non-conservative Lys to Glu variation at the 323rd position of pMGF 505-9R (Lys-323-Glu). Region II contains one unique non-conservative substitution, the Ala to Thr change at position 132 of the A238L-encoded protein (Ala-132-Thr).

Region III consists of one conservative mutation wherein asparagine was changed to serine at the 414th position of pNP419L (Asn-414-Ser). The same amino acid mutation was observed in the same position of the protein in the other isolates. Region IV consists of two mutations comprising one substitution and one insertion. A shared non-conservative isoleucine to phenylalanine substitution is observed in the 195th position of the protein pI267L encoded by the gene I267L (Ile-195-Phe). An insertion of glutamine is seen at position 35 of pDP60R (Ins-35-Glu), and this substitution is shared with the other Asian strains except PHL/StrainA4 from the Philippines (Table 5).

### 3.7. Intergenic Variations in TH1_22/CR

#### Variation in TRS

**Figure 5:**
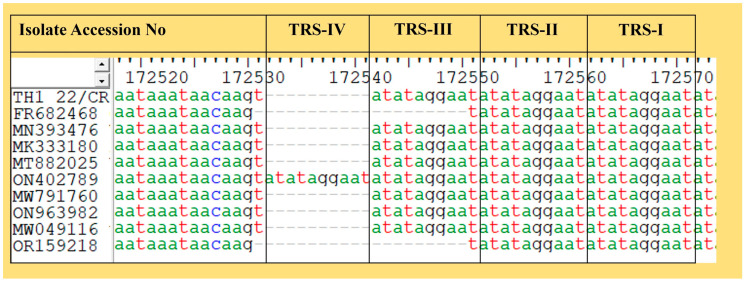
Characterization of TH1_22/CR based on the number of tandem repeat sequences. Three types of IGR types were identified in the Asian isolates out of which TH1_22/CR was characterized as TRS type-II.

In the current study, three types of IGR (IGR-I, IGR-II, and IGR-III) were observed between the I73R and I329L genes, based on the 10 bp tandem repeat sequence or TRS (ATATAGGAAT). IGR-I was represented by the Georgia reference strain (FR682468) and Korean isolate (OR159218), which contained only one copy of this TRS. One Vietnamese isolate (ON402789) was IGR-III, containing four copies of this TRS. The remaining strains, including TH1_22/CR, contained three copies of this TRS and are IGR-II (Figure 5).

### 3.8. Oligonucleotide Indels in TH1_22/CR

Our Thai isolate shows a 9 bp deletion in the IGR between the 110-13La and 110-13Lb genes which leads to the deletion of 20 bp at the 5’ end of the 110-13Lb gene. A 52 bp deletion was also found in the ORF of the ASFV G ACD 00350 gene. (Table 6).

## 4. Discussion

The current study involves the strain TH1_22/CR from an ASF outbreak in Thailand. The genome of this strain was found to contain 193 potential ORFs and 185 coding sequences using genome annotation. To determine the genotype of this strain, an ML tree was constructed using publicly available complete p72 sequences (Figure 2). From the clustering of the TH1_22/CR genome observed alongside other Georgia 2007/1-originated Asian genotype II viruses, the widespread distribution of genotype II ASFV in these regions can be indicated [20,21,24,35]. As CD2v (EP402R) has been reported to be related to hemadsorption, serogrouping using the phylogenetic clustering of the EP402R gene with different strains demonstrated that our strain is antigenically related to serogroup 8 (Figure 3) [36]. The CVR analysis revealed TH1_22/CR to be completely similar to CVR-I Georgia 2007/1 due to the presence of the “BNDBNDBNAA” TRS sequence [37].

It has been discovered that ASFV is transmitted to other Asian countries through China [38]. Therefore, TH1_22/CR was examined using ASFV strains from countries including China, Vietnam, the Philippines, and Korea, obtained from the NCBI public database (Figure 4). Two representatives out of the whole genome sequences from each country were chosen, along with the reference strain Georgia 2007/1, for the variation analysis and similarity index of TH1_22/CR.

Our assessments reveal a considerable degree of similarity across the entire genomes of TH1_22/CR, Georgia 2007/1 (99.9% ANI), and the strains from China (Wuhan 2019-1 and Pig/HLJ/2018), the Philippines (PHL/Strain A4 and ASFV2019-003-B), Korea (Korea/pig/Yeoncheon1/2019 and S-S-VR-413000-00008), and Vietnam (VN/2019 and VN/2021). The range of 99.1% to 99.9% reveals a close genetic relationship between our strain and the other Georgia 2007/1-originated Asian genotype II genomes, indicating that region-wise mutations in different countries could influence the variations in similarity observed (Table 2).

Out of the 195 genes analyzed in all the considered isolates, 27 genes had variations in their ORFs (Figure 5, Table 2). All the isolates, including our Thai strain, were reported with a total of 39 SNPs and Indels in 28 ORFs, i.e., n = 16 SNPs and n = 23 indels. For ease of analysis, the entire ASFV genome (based on the reference Georgia 2007/1) was divided into Regions I to IV. As a result, TH1_22/CR was observed to harbor two unique mutations and six shared variations on a genetic and amino acid level throughout these regions (Figure 4 and Figure 5).

TH1_22/CR reports two unique mutations in the ORFs of its genome. The C-591-T and Ala-192-Thr variations can be seen in the MGF 360-4L gene, whose exact function has not been understood yet. A238L also reports unique variations in C-297-T and Ala-132-Thr. Since A238L has been reported to downregulate TNF-α and COX-2, this variation may affect the virulence of this strain [39,40]. The shared C-51-T nucleotide change and the Pro-19-Leu change are seen in MGF 110-1L. This gene might aid in the survival of the virus as it is the sole MGF 110 gene present in all ASFV genomes [41]. For MGF 360-10L, the T-53-C and Val-18-Ala variations have been observed. Since this gene can degrade JAK1 through K48-linked ubiquitination, this mutation could aid our virus’s ability to suppress interferon-stimulated genes or ISGs [42].

MGF 505-9R has the mutations of A-962-G and the non-conservative Lys-323-Glu. While the specific function of MGF505-9R is not yet understood, since it is widely recognized that MGF530/505 genes play a crucial role in inhibiting the initiation of the type I IFN pathway, this SNP could potentially aid in virulence [43]. T-222-A and the non-conservative Ile-195-Phe are observed in I267L, which could enhance the virulence of the strain as this gene has been recognized to have multi-faceted actions of inhibiting RNA Pol-III-RIG-I signaling, suppressing IFN-β production, modulating hemorrhages, etc. [44,45]. The other variations are the C-20-T and Asn-414-Ser changes in the DNA ligase-encoding NP419L gene, and Ins-104-A and Ins-35-Glu in DP60R. The exact virulence mechanisms of these genes have not been elucidated yet.

ASFV, being a DNA virus, possesses a 3′ to 5′ exonuclease proofreading enzyme, probably evolved to adapt to the oxidative stress created by macrophages, thereby playing a role in the limited occurrence of single SNPs [46]. The virus includes a versatile DNA ligase that could potentially aid in creating indels via non-homologous end-joining. This could explain the predominant pattern in distinguishing Thai genotype II viruses from other strains to be indels rather than SNPs, but the prevalence of this mode of genome evolution is not completely understood.

Interestingly, the variations in oligonucleotides were mostly found in Region I, either in introns or exons, with no variation recorded in Region II, III, and IV. One hypothesis posits that such discrepancies may stem from sequencing errors as contemporary sequencing techniques encounter challenges in accurately resolving such mutations due to their inherent limitations. Alternatively, it could signify intra-strain sequence variation resulting from replication slippage within nucleotide tracts [35,47]. Variability within tandem repeat sequences, particularly in the CVR and the IGR of I73R/I329R, has been documented as an attempt at differentiating and characterizing ASFV isolates exploited to augment the discriminatory capabilities of ASFV isolates [48].

## 5. Conclusions

The Thai isolate of ASFV, TH1_22/CR, was submitted to GenBank (Accession No. PP915735). Based on the phylogenetic analysis, the Thai ASFV strain TH1_22/CR was confirmed to belong to genotype II, serogroup 8, and IGR-II. This strain is 189,323 bp long, containing 193 ORFs, and it shares the highest similarity with ASFV detected in Pig/HLJ/2018 and Wuhan 2019-1. In the Thai isolate, eight SNPs and indels were reported, which include two unique SNPs. Furthermore, a 12 bp unique intergenic region (IGR) was found deleted between the 110-13La and 110-13Lb genes. Overall, in Asia, a total of 39 SNPs and indels were reported from Asian isolates in 28 genes. About 52 nucleotides of one gene were deleted in the ASFV G ACD 00350 genes. Determining whether all genotype II ASFV strains share a precise "core number" of coding sequences, however, is challenging due to the incomplete annotation of many genomes in public databases.

## Figures and Tables

**Table 1:** Full-length genotype II Asian ASFV strains retrieved from NCBI.

No.	Accession No.	Isolate Name	City	Country	Genotype	Year	Genome	Length (bp)
1	FR682468	Georgia 2007/1	Georgia	Georgia	II	2007	Full-length	190,584
2	MK333180	HLJ/2018	Harbin, Heilongjiang	China	II	2018	Full-length	189,404
3	MN393476	Wuhan 2019-1	Wuhan, Hubei	China	II	2019	Full-length	190,576
4	MK128995	Anhui/XCGQ	Qingdao, Shandong	China	II	2018	Full-length	189,393
5	OP856591	LN/2018/1	Qingdao, Shandong	China	II	2018	Full-length	189,397
6	MT496893	GZ201801	Guangzhou, Guangdong	China	II	2018	Full-length	189,393
7	MK645909	wbBS01	Changchun, Jilin	China	II	2018	Full-length	189,394
8	MZ614662	CADC HN09	Beijing	China	II	2019	Full-length	190,257
9	MN172368	CAS19-01	Wuhan, Hubei	China	II	2019	Full-length	189,405
10	MW033528	wbShX01	Changchun, Jilin	China	II	2019	Full-length	189,401
11	MK940252	InnerMongolia-AES01	Changchun, Jilin	China	II	2019	Full-length	189,403
12	OM161110	SY-1	Wuhan, Hubei	China	II	2020	Full-length	189,404
13	MW521382	HuB20	Changchun, Jilin	China	II	2020	Full-length	188,643
14	MW656282	Heilongjiang/HRB1/2020	Harbin, Heilongjiang	China	II	2020	Full-length	189,355
15	OL310288	China/2020	Lanzhou, Gansu	China	II	2020	Full-length	188,072
16	OP612151	SY-2	Wuhan, Hubei	China	II	2021	Full-length	189,404
17	ON456300	Yangzhou	Yangzhou	China	II	2021	Full-length	187,951
18	MT882025	VN/QP-ASFV1(2019)	Hanoi	Vietnam	II	2019	Full-length	189,081
19	ON402789	VN/2021	Ninh Kieu District, Can Tho	Vietnam	II	2021	Full-length	189,487
20	MW049116	Yeoncheon1/2019	Gimcheon, Gyeongsangbuk-do	Korea	II	2019	Full-length	187,848
21	OR159219	S-S-VR-413000-00002	Ik-San, Jeollabuk-Do	Korea	II	2019	Full-length	189,400
22	OR159218	S-S-VR-413000-00008	Ik-San, Jeollabuk-Do	Korea	II	2019	Full-length	189,429
23	MT748042	PaJu1/2019	Gimcheon, Gyeongsangbuk-do	Korea	II	2019	Full-length	187,848
24	OR159217	S-S-VR-413000-00015	Ik-San, Jeollabuk-Do	Korea	II	2019	Full-length	189,411
25	MW791752	ASFV2020-008-B	Manila	Philippines	II	2020	Full-length	190,565
26	MW791759	ASFV2020-021-B	Manila	Philippines	II	2020	Full-length	190,571
27	MW791760	ASFV2019-003-B	Manila	Philippines	II	2019	Full-length	190,571
28	MW791761	ASFV2020-003-B	Manila	Philippines	II	2020	Full-length	190,571
29	ON963982	PHL/Strain A4	Los Banos, Laguna	Philippines	II	2021	Full-length	192,265
30	MW396979	Timor-Leste/2019/1	Timor-Leste	Timor-Leste	II	2019	Full-length	192,237
31		TH1_CR/2022	Chiang Rai	Thailand	II	2022	Full-length	189,395

**Table 2:** Similarity identity matrix of the nucleotide sequences (highlighted in green) and amino acid sequences (highlighted in orange) of the ASFV strains. The analysis shows that TH1_22/CR has maximum similarity (99.9%) with Georgia 2007/1 and the Chinese (Wuhan 2019-1 and China HLJ) isolates.

Sequence Identity Matrix	TH1_22/CR_Thailand	FR682468.2 Georgia 2007/1_Georgia	MN393476_Wuhan/2019-1_China	MK333180_HLJ_China/2018	MT882025.1 VN/QP-ASFV1(2019)_Vietnam	ON402789_Serotype 8 Genotype 2 (VN/2021)_Vietnam	MW791760_2019-003-B_2019/Philippines	ON963982_PHL/StrainA4_Philippines (2021)	MW049116_Pig/Yeoncheon1/2019_Korea (2019)	OR159218_S-S-VR-413000-00008_Korea (2019)
TH1_22/CR_Thailand	ID	99.9	99.9	99.9	99.7	99.2	99.9	99.9	99.1	99.9
FR682468.2 Georgia 2007/1_Georgia	99.9	ID	99.9	99.9	99.7	99.2	99.9	99.9	99.1	99.9
MN393476_Wuhan/2019-1_China	99.9	99.9	ID	99.9	99.7	99.2	99.9	99.9	99.1	99.9
MK333180_HLJ_China/2018	99.9	99.9	99.9	ID	99.7	99.2	99.9	99.9	99.1	99.9
MT882025.1 VN/QP-ASFV1(2019) Vietnam	99.6	99.6	99.6	99.6	ID	99	99.7	99.7	98.9	99.7
ON402789_Serotype 8 genotype 2 (VN/2021) Vietnam	99.2	99.2	99.2	99.2	98.9	ID	99.2	99.2	99.6	99.2
MW791760_2019-003-B_2019/Philippines	99.9	99.9	99.9	99.9	99.6	99.2	ID	99.9	99.1	99.9
ON963982_PHL/StrainA4_Philippines (2021)	99.8	99.8	99.9	99.9	99.6	99.1	99.9	ID	99.1	99.9
MW049116_pig/Yeoncheon1/2019_Korea (2019)	99.1	99.1	99.1	99.1	98.8	99.6	99.1	99	ID	99.1
OR159218_S-S-VR-413000-00008_Korea (2019)	99.8	99.9	99.9	99.9	99.6	99.2	99.9	99.8	99.1	ID

**Table 3:** The distribution of variable genes across the ASFV genome in Asian strains. The whole genome is divided into four regions to understand the localization of a variable gene in the genome. Percentage = (No. of variable genes/Total genes in region) × 100.

Regions	Total Genes in Region	No. of Variable Genes	Percentage	Genes Variable in Asia
Region-I (From 1 to 47,170 bp)	59	11	18.64	MGF 360-2L MGF 110-1L MGF 110-3L MGF 110-7L MGF 360-4L MGF 360-10L MGF 300-2R MGF 505-1R MGF 360-14L MGF 505-4R MGF 505-9R
Region-II (From 47,171 to 101,665 bp)	49	7	14.2	A238L EP364R F165R EP153R M1249L C84L C717R
Region-III (From 101,666 to 162,619 bp)	30	5	16.66	G1211R O174L NP1450L NP419L D1133L
Region-IV (From 162,620 to 190,169 bp)	57	5	8.77	I267L MGF-360 16R I10L ASFV G ACD 1980 DP60R
Total	195	28	14.35	

**Table 4:** Genetic variation in the ASFV genomes on a nucleotide level in Region I, Region II, Region III, and Region IV. The green bars under the table represent the regions. The blue-highlighted cells represent variations in TH1_22/CR. The yellow-highlighted cells indicate variations in the other strains.

Gene Name	MGF 360-2L	MGF 110-1L	MGF 110-3L					MGF 110-7L	MGF 360-4L		MGF 360-10L		MGF 300-2R	MGF 505-1R	MGF 360-14L		MGF 505-4R	MGF 505-9R
**Regions**	**Region I**																	
**Location in Gene**	**365**	**51**	**42**	**235**	**236**	**249**	**328**	**365**	**438**	**591**	**53**	**811**	**216**	**1155**	**224**	**250**	**1020**	**962**
**FR682468.2** **Georgia 2007/1_Georgia**	A	C	G	G	T	A	A	C	C	C	T	G	A	G	X	T	T	A
**TH1_22/CR** **Thailand (2022)**	A	T	G	G	T	A	A	C	C	T	C	G	A	G	X	T	T	G
**MN393476** **Wuhan/2019-1_China**	A	T	G	G	T	A	A	C	C	C	C	G	A	G	C	T	T	G
**MK333180** **HLJ_China/2018**	A	T	G	G	T	A	A	C	C	C	C	G	A	G	X	T	T	G
**MT882025.1** **VN/QP-ASFV1(2019)_Vietnam**	A	T	G	C	C	A	A	C	C	C	C	G	A	G	C	T	T	G
**ON402789_Serotype 8 genotype 2 (VN/2021)_Vietnam**	X	T	A	C	C	G	C	X	T	C	C	G	A	G	X	X	T	G
**MW791760** **2019-003-B_2019/Philippines**	X	T	G	G	T	A	A	X	C	C	C	G	A	G	X	T	T	G
**ON963982** **PHL/StrainA4_Philippines(2021)**	X	T	G	G	T	A	A	X	C	C	C	G	X	X	X	T	X	G
**MW049116** **pig/Yeoncheon1/2019_Korea(2019)**	A	T	G	G	T	A	A	C	C	C	C	C	A	G	X	T	T	G
**OR159218** **S-S-VR-413000-00008_Korea(2019)**	A	T	G	G	T	A	A	C	C	C	C	C	A	G	X	T	T	G
**Gene Name**	A238L	EP364R		F165R	EP153R	M1249L	C84L	C717R	G1211R	O174L	NP1450L		NP419L		D1133L			
**Regions**	**Region II**								**Region III**									
**Location in Gene**	**297**	**791**		**69**	**52**	**3461**	**156**	**797**	**3296**	**128**	**926**		**20**	**935**	**278**	**1366**	**3241**	
**FR682468.2** **Georgia 2007/1_Georgia**	C	T		T	T	T	T	T	A	T	T		C	C	T	G	C	
**TH1_22/CR** **Thailand (2022)**	T	T		T	T	T	T	T	A	T	T		T	C	T	G	C	
**MN393476** **Wuhan/2019-1_China**	C	T		T	T	T	T	G	A	T	T		T	C	T	G	C	
**MK333180** **HLJ_China/2018**	C	T		T	T	T	T	T	A	T	T		T	C	X	G	C	
**MT882025.1** **VN/QP-ASFV1(2019)_Vietnam**	C	T		T	T	T	T	T	A	T	T		T	T	T	G	C	
**ON402789_Serotype 8 genotype 2 (VN/2021)_Vietnam**	C	T		T	T	T	T	T	A	T	T		T	C	T	G	C	
**MW791760** **2019-003-B_2019/Philippines**	C	T		T	T	T	T	T	A	T	T		T	C	T	G	C	
**ON963982** **PHL/StrainA4_Philippines(2021)**	C	T		X	X	X	X	T	X	X	X		T	C	T	X	X	
**MW049116** **pig/Yeoncheon1/2019_Korea(2019)**	C	X		T	T	T	T	T	A	T	T		T	C	T	G	C	
**OR159218** **S-S-VR-413000-00008_Korea(2019)**	C	T		T	T	T	T	T	A	T	T		T	C	T	G	C	
**Gene Name**	**I267L**	**MGF 360-16R**				**I10L**		**ASFV G ACD 01980**	**DP60R**		**NCR**		**NCR**		**NCR**		**NCR**	
**Regions**	**Region IV**																	
**Location in Gene**	**222**	**260**		**898**		**415**		**179**	**104**		**189,406**		**189,433**		**189,439**		**189,459**	
**FR682468.2** **Georgia 2007/1_Georgia**	T	G		A		T		T	X		A		C		G		T	
**TH1_22/CR** **Thailand (2022)**	A	G		A		T		T	A		G		G		A		A	
**MN393476** **Wuhan/2019-1_China**	A	G		A		T		T	A		A		C		G		T	
**MK333180** **HLJ_China/2018**	A	G		A		T		T	A		A		C		G		T	
**MT882025.1** **VN/QP-ASFV1(2019)_Vietnam**	A	G		A		T		T	A		A		C		G		T	
**ON402789_Serotype 8 genotype 2 (VN/2021)_Vietnam**	A	G		A		T		T	A		A		C		G		T	
**MW791760** **2019-003-B_2019/Philippines**	A	G		A		T		T	A		A		C		G		T	
**ON963982** **PHL/StrainA4_Philippines(2021)**	A	X		X		X		X	X		A		C		G		T	
**MW049116** **pig/Yeoncheon1/2019_Korea(2019)**	A	G		A		T		T	A		A		C		G		T	
**OR159218** **S-S-VR-413000-00008_Korea(2019)**	A	G		A		T		T	A		A		C		G		T	

**Table 5:** Genetic variation in the ASFV genomes on an amino acid level in Region I, Region II, Region III and Region IV. The green bars under the table represent the regions. The blue-highlighted cells represent variations in TH1_22/CR. The yellow-highlighted cells indicate variations in the other strains.

Gene Name	MGF 360-2L	MGF 110-1L	MGF 110-3L			MGF 110-7L	MGF 360-4L		MGF 360-10L		MGF 300-2R	MGF 505-1R	MGF 360-14L		MGF 505-4R	MGF 505-9R
**Regions**	**Region I**															
**Location in Gene**	**242**	**19**	**18**	**47**	**112**	**13**	**192**	**243**	**18**	**271**	**72**	**282**	**276**	**184**	**340**	**323**
**FR682468.2** **Georgia 2007/1_Georgia**	**Leu**	Pro	Leu	Asp	His	Ala	Ala	Val	Val	Glu	Ile	Ala	Met	X	Leu	Lys
**TH1_22/CR** **Thailand (2022)**	**Leu**	Leu	Leu	Asp	His	Ala	Thr	Val	Ala	Glu	Ile	Ala	Met	X	Leu	Glu
**MN393476** **Wuhan/2019-1_China**	**Leu**	Leu	Leu	Asp	His	Ala	Ala	Val	Ala	Glu	Ile	Ala	Met	Met	Leu	Glu
**MK333180** **HLJ_China/2018**	**Leu**	Leu	Leu	Asp	His	Ala	Ala	Val	Ala	Glu	Ile	Ala	Met	X	Leu	Glu
**MT882025.1** **VN/QP-ASFV1(2019)Vietnam**	**Leu**	Leu	Leu	Asp	His	Ala	Ala	Val	Ala	Glu	Ile	Ala	Met	Met	Leu	Glu
**ON402789** **VN/2021_Vietnam**	**X**	Leu	Val	Arg	Tyr	X	Ala	Ile	Ala	Glu	Ile	Ala	X	X	Leu	Glu
**MW791760** **2019-003-B_2019/Philippines**	**X**	Leu	Leu	Asp	His	X	Ala	Val	Ala	Glu	Ile	Ala	Met	X	Leu	Glu
**ON963982** **PHL/StrainA4_Philippines(2021)**	**X**	Leu	Leu	Asp	His	X	Ala	Val	Ala	Glu	X	X	Met	X	X	Glu
**MW049116** **pig/Yeoncheon1/2019_Korea(2019)**	**Leu**	Leu	Leu	Asp	His	Ala	Ala	Val	Ala	Glu	Ile	Ala	Met	X	Leu	Glu
**OR159218** **S-S-VR-413000-00008_Korea(2019)**	**Leu**	Leu	Leu	Asp	His	Ala	Ala	Val	Ala	Glu	Ile	Ale	Met	X	Leu	Glu
**Gene Name**	A238L	EP364R		F165R	EP153R	M1249L	C84L	C717R	G1211R	O174L	NP419L		D1133L			
**Regions**	**Region II**								**Region III**							
**Location in Gene**	**132**	**264**		**23**	**18**	**92**	**26**	**266**	**1099**	**133**	**109**	**414**	**54**	**679**	**1042**	
**FR682468.2** **Georgia 2007/1_Georgia**	Ala	Leu		Asn	Lys	Lys	Arg	Val	Glu	Lys	Arg	Asn	Gly	Pro	Gln	
**TH1_22/CR** **Thailand (2022)**	Thr	Leu		Asn	Lys	Lys	Arg	Val	Glu	Lys	Arg	Ser	Gly	Pro	Gln	
**MN393476** **Wuhan/2019-1_China**	Ala	Leu		Asn	Lys	Lys	Arg	Glu	Glu	Lys	Arg	Ser	Gly	Pro	Gln	
**MK333180** **HLJ_China/2018**	Ala	Leu		Asn	Lys	Lys	Arg	Val	Glu	Lys	Arg	Ser	Gly	Pro	X	
**MT882025.1** **VN/QP-ASFV1(2019)Vietnam**	Ala	Leu		Asn	Lys	Lys	Arg	Val	Glu	Lys	His	Ser	Gly	Pro	Gln	
**ON402789** **VN/2021_Vietnam**	Ala	Leu		Asn	Lys	Lys	Arg	Val	Glu	Lys	Arg	Ser	Gly	Pro	Gln	
**MW791760** **2019-003-B_2019/Philippines**	Ala	Leu		Asn	Lys	Lys	Arg	Val	Glu	Lys	Arg	Ser	Gly	Pro	Gln	
**ON963982** **PHL/StrainA4_Philippines(2021)**	Ala	X		X	X	X	X	Val	X	X	Arg	Ser	X	X	Gln	
**MW049116** **pig/Yeoncheon1/2019_Korea(2019)**	Ala	Leu		Asn	Lys	Lys	Arg	Val	Glu	Lys	Arg	Ser	Gly	Pro	Gln	
**OR159218** **S-S-VR-413000-00008_Korea(2019)**	Ala	Leu		Asn	Lys	Lys	Arg	Val	Glu	Lys	Arg	Ser	Gly	Pro	Gln	
**Gene Name**	I267L			MGF 360-16R					I10L		ASFV G ACD 01980				DP60R	
**Regions**	**Region IV**															
**Location in Gene**	**195**			**87**		**300**			**91**		**60**		**238**		**35**	
**FR682468.2** **Georgia 2007/1_Georgia**	Ile			Trp		Lys			Asn		Ile		Lys		X	
**TH1_22/CR** **Thailand (2022)**	Phe			Trp		Lys			Asn		Ile		Lys		Gln	
**MN393476** **Wuhan/2019-1_China**	Phe			Trp		Lys			Asn		Ile		Lys		Gln	
**MK333180** **HLJ_China/2018**	Phe			Trp		Lys			Asn		Ile		Lys		Gln	
**MT882025.1** **VN/QP-ASFV1(2019)Vietnam**	Phe			Trp		Lys			Asn		Ile		Lys		Gln	
**ON402789** **VN/2021_Vietnam**	Phe			Trp		Lys			Asn		Ile		Lys		Gln	
**MW791760** **2019-003-B_2019/Philippines**	Phe			Trp		Lys			Asn		Ile		Lys		Gln	
**ON963982** **PHL/StrainA4_Philippines(2021)**	Phe			X		X			X		X		X		X	
**MW049116** **pig/Yeoncheon1/2019_Korea(2019)**	Phe			Trp		Lys			Asn		Ile		Lys		Gln	
**OR159218** **S-S-VR-413000-00008_Korea(2019)**	Phe			Trp		Lys			Asn		Ile		Lys		Gln	

**Table 6:** Deletion of oligonucleotides in TH1_22/CR: a 29 bp deletion in IGR between 110-13La- and 110-13Lb genes and a 52 bp deletion in ASFV G ACD 00350 gene in the TH1_22/CR genome.

S. No.	Gene Name	Indels	Isolate Name
1	110-13La-IGR-110-13Lb	29 bp deletion	TH1_22/CR
2	ASFV G ACD 00350	52 bp deletion	TH1_22/CR
3	EP402R	18 bp deletion	Vietnam (ON402789)
4	B475L	78 bp addition	Vietnam (ON402789)

## Data Availability

All data analyzed during this study are included in this published article and Supplementary Materials. The raw data are available from the corresponding author upon reasonable request.

## Supplementary Materials

The following supporting information can be downloaded at https://www.mdpi.com/article/10.3390/ani14172602/s1, Table S1: B646L (p72) gene sequences of ASFV strains with their accession numbers for phylogenetic tree construction.
