# Supplementary material for: The Comparative Full-Length Genome Characterization of African Swine Fever Virus Detected in Thailand
Source: Animals (Basel). 2024 Sep 6;14(17):2602. doi: 10.3390/ani14172602 (PMC11394130; doi:10.3390/ani14172602)
Supplement: Supplementary file 1 [file animals-14-02602-s001.zip › animals-3157632-supplementary.pdf]

Table S1: B646L (p72) gene sequences of ASFV strains for Phylogenetic tree construction

No	Isolate	Accession No	p72 genotype	Year of isolation	Country
1	Ndjassi-77	KM236553	I	1979	Zaire (DRC)
2	Lisbon/57	AF301537	I	1957	Portugal
3	China/Guangxi/2019	MK670727	II	2019	China
4	CN201801	MH722357	II	2018	China
5	China/Jilin/2018/boar	MK189456	II	2018	China
6	Korea/Pig/Paju1/2019	MN603967	II	2019	South Korea
7	VN/Pig/TH/411	MK554698	II	2019	Vietnam
8	IND/AS/SD-02/2020	MT612961	II	2020	India
9	Belgium/2018/Etalle	MH998358	II	2018	Belgium
10	Estonia 2014	LS478113	II	2014	Estonia
11	Bel13/Grodno	KJ627214	II	2013	Belarus
12	Georgia 2007/1	FR682468	II	2007	Georgia
13	ZAM/2017/Mbala/1	LC322016	II	2017	Zambia
14	ZIM/2015/01	KX090923	II	2015	Zimbabwe
15	BOT/1/99	AF504886	III	1999	Botswana
16	RSA/1/99/W	AF449477	IV	1999	South Africa
17	MK200	KJ526369	V	1978	Mozambique
18	MOZ/94/1	AF270711	VI	1994	Mozambique
19	SPEC/260	DQ250121	VII	1993	South Africa
20	Malawi/1978	AF270707	VIII	1978	Malawi
21	UGA2003/1	AY351564	IX	2003	Uganda
22	Hinde/1	MK829709	X	1954	Kenya
23	KAB/62	AY351522	XI	1983	Zambia
24	MFUE 6/1	AY351561	XII	1982	Zambia
25	SUM/1411	AY351542	XIII	1983	Zambia
26	NYA/12	AY351555	XIV	1986	Zambia
27	TAN/1/01	AY494552	XV	2001	Tanzania
28	TAN/2003/2	AY494551	XVI	2003	Tanzania
29	ZIM/92/1	DQ250119	XVII	1992	Zimbabwe
30	NAM/1/95	DQ250122	XVIII	1995	Namibia
31	SPEC/125	DQ250118	XIX	1987	South Africa
32	RSA/1/95	DQ250123	XX	1995	South Africa
33	RSA/1/96	DQ250125	XXI	1996	South Africa
34	SPEC/245	DQ250117	XXII	1992	South Africa
35	ETH/1	KT795354	XXIII	2011	Ethiopia
36	MOZ_10/2006	KY353989	XXIV	2006	Mozambique
